# Strengthening education in rehabilitation: Assessment technology and digitalization

**DOI:** 10.3389/fresc.2022.883270

**Published:** 2022-08-24

**Authors:** Cristina Herrera-Ligero, Joaquim Chaler, Ignacio Bermejo-Bosch

**Affiliations:** ^1^ Biomechanics Institute of Valencia, Polytechnic University of Valencia, Valencia, Spain; ^2^ University School of Health and Sport (EUSES & ENTI), University of Girona and University of Barcelona, L'Hospitalet de Llobregat, Catalonia, Spain; ^3^ PM&R Department, Hospital Egarsat, Barcelona, Spain

**Keywords:** rehabilitation, education, assessment, technologies, digitalization

## Abstract

Rehabilitation is a discipline increasingly growing around the world due to several reasons, but probably the most important one is aging population and chronicity. A need to harmonize education has been identified, and although several International organizations such as the European Union of Medical Specialists (UEMS) and the International Society of Physical Medicine and Rehabilitation (ISPRM) have defined standards, given the quick growth of new evidence and assessment methods an urge to establish new ones arises. Functional assessment and tools used to do so are key in rehabilitation processes. This comprises self-reported questionnaires, conventional clinical evaluation but more notably high technology assessment methods, such as movement analysis systems, posturography, different types of dynamometers and kinesiologic electromyography among others. More recently, a wide range of wearable systems has been introduced in patient assessment. This is generating many published protocols as well as reliability and validity studies. The objective of this narrative review is to present main assessment technologies relevant to rehabilitation, its situation of this specific area in pre-graduate and post-graduate rehabilitation educational programs, and to elaborate a formative proposal including technological foundations of assessment and also highlighting the importance of solid reliability and validity of assessment methods comprehension. The main objective of this proposal is to provide basic knowledge about rehabilitation and methodologies for outcomes evaluation, including new technologies, to all health professionals, but especially to those who work or will work in the field of Rehabilitation.

## Introduction

Rehabilitation, according to the World Health Organization (WHO), is the set of interventions designed to optimize function and reduce disability in people with health status conditions in relation to their environment. The conditions can refer to acute or chronic diseases, dysfunctions, injuries or trauma, or to other circumstances such as pregnancy, advanced age, stress, congenital anomalies or genetic predispositions (1). Anyone with a medical condition who experiences some form of function limitation such as mobility, vision, or cognitive ability may require rehabilitation. Due to the demographic evolution of most countries, marked by the increase in life expectancy and chronicity, it has been estimated that the population likely to require some rehabilitation intervention in the world is 2,410,000,000 people, therefore, this discipline challenges all health professionals, beyond specialized physicians. Rehabilitation consists of interventions aimed at addressing deficits, activity limitations, and participation restrictions, as well as personal and environmental factors (including technical assistance) that have an impact on function. To design these interventions, it is essential to define individualized objectives for each patient.

Both for the design of the best intervention and for outcomes measurement in Rehabilitation, it is essential to carry out a correct and complete function (namely bodily functions as described by ICF) evaluation of each patient. The concept of function and its evaluation refer to the capacity of the person to carry out activities or tasks necessary for self-care and interaction with the environment, as well as the measure of that capacity. It is a broad concept, which encompasses the physical, mental, affective and social spheres of the person (2). These functional evaluation skills must be part of the rehabilitation professional's own competences. The accuracy of assessments will determine whether available resources are used with sustainability and effectiveness, establishing concrete and personalized objectives in each case and achieving the best possible result in terms of structure, function, activity and participation of the person (3).

Among the instruments for the measurement of functional capacity, the assessment scales or Patient Reported Outcome Measurements (PROMs) have had a great development in the last 60 years, being widely known and used by rehabilitation professionals. The usefulness of such tools has been widely demonstrated and they have been found to slightly improve quality of life and increase communication between patients and their doctors (4).

Despite the usefulness and multiple advantages of these tools, there is no general consensus on which to use ideally in each case, and there may be problems with the choice, interpretation and use of the information of the scales for rehabilitation processes. On the other hand, we must ensure that they are validated, reliable and adapted to the local language, ideally through a cross-cultural adaptation process (2). In addition, in most cases they are not exempt from some component of subjectivity, arising from the tested patient or the tester. Such gaps make it essential to confront results from clinical scales with objective body function data, like those obtained by means of appropriate biomechanical assessment technologies.

For years, a wide variety of tools and technologies for biomechanical and functional assessment have emerged. These techniques approach functional assessment in a quantitative and objective way, focusing on the analysis of the physical sphere of different human functions and activities. The use of different kind of technologies enable the study of physical parameters during a given activity, like gait or reaching (5) or while performing any function, like balance or muscle power function. Those parameters may be related to different aspects, highlighting the following:

• How the movement is performed: kinematic properties refer to movement quality, and include parameters like range of motion, speed or acceleration. These might be measured with varied tools of different complexity, from classic goniometers, to electrogoniometers, inertial sensors or photogrammetry in 2, 3, and even 4 dimensions. Not every technique allows registering every kind of parameters, as some may only measure range of motion or speed.
• Causes of movement and muscle power function: forces are in turn responsible for movements. In this case, measurement tools can focus on the analytical study of muscle function through isometric and isokinetic dynamometry, or on the study of other types of variables such as ground reaction forces through dynamometric platforms, widely used for the evaluation of human gait and also the basis of posturography.
• In the assessment of physiological signals, the use of electromyography, mainly surface, for the analysis of muscle activation during activities such as walking is relatively widespread. There are also other types of techniques for the analysis of physiological signals such as heart rate, blood pressure, respiratory volumes or tissue temperature, among many others.

Though some of these techniques have been classically located within a movement analysis lab, in recent years a wide range of simple wearable systems and smartphone-based tools have arisen, allowing an objective register of daily overall or specific activity of patients not only within clinical settings, but also in the community.

The clinical utility of biomechanical analysis technologies has been proven in different areas. One of the most evaluated activities has been gait, for which biomechanical analysis has been related to greater confidence in decision-making, change in decisions and an improve of the agreement between clinicians (6). A high degree of evidence has been proven either in the relationship between biomechanical analysis and better functional outcomes in populations such as children with cerebral palsy or adults with acquired brain injury, both in relation to surgical treatment and non-surgical management. In relation to the above, the performance of this type of analysis can lead to a saving of resources in children with cerebral palsy (7).

Another of the most analyzed activities is balance, through the well-known posturography (static or dynamic). This analysis technique has been used for years, both for functional assessment and for the treatment of balance disorders, and among other achievements it has allowed the detailed description of the pathophysiology of different pathologies that affect balance (8). Its clinical usefulness has also been proven for the evaluation and identification of specific populations, such as those with dizziness of cervicogenic origin (9).

As for dynamometry, isometric grip (namely Jamar) dynamometers (10), isokinetic dynamometry, the “gold standard” of dynamometry assessment (11–13), and hand-held dynamometry (14, 15), show a growing evidence of reliability and validity in several clinical situations. For instance, at this moment grip strength Jamar dynamometer test is the first criteria to detect sarcopenia (16).

To be truly adequate for its use in clinical practice, any of the above-mentioned tools should be reliable, valid and with high responsiveness. Reliability is the property that indicates that the measurement offers equivalent results when carried out under similar conditions. This property is evaluated through inter-tester reliability studies or in test-retest studies. Reliability is an essential prerequisite to render a test valid. On the other hand, validity is understood as the property that indicates that the measure really represents the aspect to be evaluated, that is, the correspondence between what is measured and the reality that is to be represented. Normally, the validity of a measuring instrument is evaluated by comparing with a benchmark or gold standard. Finally, responsiveness or sensitivity is the tool capacity of detecting changes. Clinicians must know well these concepts and know how to identify them in each measurement tool, in order to always choose the most appropriate.

In addition to selecting the appropriate measurement tool, healthcare professionals must handle all existing generated data with expertise, including that obtained through a detailed functional assessment. Only by taking all the information of the person into account from a holistic view, will a true personalized medicine be possible. Given the high amount of data and information sources available, the management of these can become extremely complex, with the associated risk of ignoring some relevant aspect in decision making. In this context, both the rise of techniques such as Big Data and Thick Data, which allow the efficient management of large volumes of qualitative and quantitative data, and the advancement of different modalities of Artificial Intelligence for the analysis and interpretation of such data, can facilitate and get the most out of all the available information. In turn, this leads to the design of algorithms aimed at improving diagnoses, assisting in decision making, or offering prognostic information, among other functionalities (17–19). These approaches have shown their effectiveness in several areas such as oncology (20), musculoskeletal injury physical therapy (21) or stroke management (22).

Considering the higher and higher importance that Artificial intelligence is reaching among clinical fields, more training for health care professionals and decision makers about its strengths, limitations and applicability is needed. This knowledge would additionally boost the adoption of these technologies by health systems, with the advantages it might bring (19). There are examples in certain areas where Artificial Intelligence has become a common reality, as is the case of medical imaging. Thus, some authors have even proposed training programs on artificial intelligence aimed at radiology medical residents (23). But training in this area, adjusted to the needs of each group, must be extended to other medical specialties and other healthcare workers.

In short, the progress of Rehabilitation as a specialty involves optimizing the ability to evaluate function, accuracy in the measurement of results and actualized skills in data management pertaining the patients. Only this way will a true valued-based and person-centered medicine be possible, considering the existing scientific evidence and supported by all the knowledge generated thanks to the advance of data recording and analysis technologies.

## Training deficits in rehabilitation

Surprisingly, despite the increasing need for rehabilitation recognized by the WHO in relation to the large increase of persons with functional deficits, the training of future physicians and other health professions suffers from significant deficits in terms of specific competences related to the discipline.

The highly increasing number of persons with disability around the world requires an active involvement of all health care professionals, beyond those dedicated exclusively to rehabilitation. That is why all doctors and healthcare professionals would require a basic knowledge about this discipline. As an example of this, among the modules considered mandatory in the curriculum of the Degree in Medicine described in Spain (24), the one related to the “Diagnostic and Therapeutic Procedures”, which comprises 40 European credits, must include, among other competences, that of [… *Know the fundamentals of rehabilitation, the promotion of personal autonomy, the functional adaptation of the environment, and other physical procedures in morbidity, for the improvement of the quality of life … *].

In spite of this, specific training programs of the Degree in Medicine of different Spanish faculties show a scarce representation of Rehabilitation. For example, in some cases it is taught as a compulsory subject but the teaching load is only 3 credits, in others it is only included as an optional subject, and on some occasions, it is not even included among this last group of subjects. This implies that many future medical professionals will graduate without basic knowledge about this subject. In line with this, a survey aimed at identifying training needs carried out by the Biomechanics Institute of Valencia, in collaboration with the Spanish (SERMEF) and Valencian (SVMEFR) Societies of Physical Medicine and Rehabilitation in 2010, showed that 28% of the 138 medical specialists rated the training received during the period of degree or bachelor's degree either insufficient or totally absent.

Needless to say, if the teaching load referred to Rehabilitation as a discipline is low, that related to the biomechanics of the musculoskeletal system and functional assessment, whose teaching is intertwined in the previous one, is even lower, being too often practically nonexistent.

In the surveys carried out within the framework of the Erasmus Plus “TEACH” project to 104 undergraduate teachers in health sciences from twelve different countries in Europe, only 32% declared that they had received some type of official training on the biomechanics of the musculoskeletal system during their university stage, a figure that drops to 17% when asked about instrumented analysis techniques. Even more striking is the fact that 46% said they were unfamiliar with the concept of functional assessment. In addition, despite the fact that 89% considered it important to include aspects related to biomechanics and instrumented analysis in the degrees of health sciences, 69% said that they are currently not taught or do so in a very insufficient way (25).

Other fundamental health profiles for any health system, such as nursing, also count on scarce training in these concepts. As an example, in the training program of the Degree in Nursing of Spain (26), although including the acquisition of skills to understand and evaluate the functioning of the person in their environment, or understand the interactive behavior of the person, no specific mention is made of the concept of function and functional assessment in all populations and pathologies, and there are no contents related to Rehabilitation or its branches. This is concerning, since nurses are a cornerstone in many programs of care, prevention, detection and treatment of any pathological and/or vital process. These professionals are involved in decision-making and have a more holistic view of people that can help detect situations where it is necessary to implement a rehabilitation intervention. Therefore, it is imperative that they know the most relevant aspects of Rehabilitation, including those related to the measurement of function.

The specialists in Physical Medicine and Rehabilitation themselves also suffer from a lack of regulated and sufficient training in functional assessment and biomechanics area. In the training program of the specialty included by the Spanish Ministry of Health (27) it is indicated that, among other aspects, the doctor specialized in training must *[… acquire adequate knowledge on biomechanics and pathomechanics of the Musculoskeletal System, as well as acquire skills in the various functional assessment systems: assessment scales, such as International Classification of Functioning (ICF)*, *American Medical Association (AMA) guidelines, Functional Independence Measurement (FIM), and instrumental methods: dynamometry, isokinetics*, *posturography, gait analysis, etc.* ]. In the same way, the training program proposed by the Panel of Physical Medicine and Rehabilitation of the European Union of Medical Specialties (UEMS), includes training in biomechanics and functional assessment, both clinical and instrumented, as well as the acquisition of skills for the management of the different evaluation methodologies (28). However, in real practice this aspect is sometimes relegated to other competences considered as more critical, remains in the hands of the preferences of the doctor in training themselves or of their mentors, or it depends on an unequal availability and access to both training resources and to the analysis methodologies.

In a survey published in 2021 (29), 77.7% of the 112 physical and rehabilitation resident doctors surveyed pointed to Biomechanics as an area of interest, and yet only 18.8% declared having sufficient training resources available. Likewise, the results of a questionnaire administered in the context of the Leonardo Da Vinci “Biomechanics4rehab” program (30) indicated that, of 184 Rehabilitation specialists from across Europe (contacted by the European Society of Physical Medicine and Rehabilitation: ESPRM), 87% had not completed any training in Biomechanics and/or analysis methodologies in the last 10 years, and that 56% would not know how to interpret the results of a biomechanical analysis.

The interest of healthcare professionals in improving their knowledge and skills in assessment, and specifically in Biomechanics, is also reflected in the demand for resources out of their formal training. An example of this is the Master of Clinical Biomechanical Assessment of the Polytechnic University of Valencia, whose number of students of different health-related profiles and from various Spanish speaking countries, rises to 207 in its 7 editions.

In brief, it appears widely proven that the training in rehabilitation, and specifically in functional assessment and biomechanics, is quite poor in health professionals and, more strikingly, in some cases of rehab professionals. Thus, a reinforcement of the body of knowledge in this important area is mandatory to strengthen the discipline all around the world.

## Discussion and proposal

Function is part of health, and as such, it must be considered in the healing or improvement of any process, whatever its origin. However, many medical specialists focus on treating only etiology, losing sight of the treatment of function, and therefore failing to maximize the quality of life of the person.

**At this moment it is clear that powerful and well-organized Rehabilitation Clinical Pathways are central in** ensuring the monitoring and treatment of the functional status of persons suffering any kind of disability. On the other hand, its efficiency and sustainability will depend on the correct assessment of function and disability. High quality information is crucial to make decisions in rehabilitation, and this will improve the results of the process, reduce the degree of disability, increase the quality of life of the population, and manage resources efficiently and coherently. In the end, this will result in greater patient and professional satisfaction.

However, training gaps can be identified at the Undergraduate and Postgraduate level that concern Rehabilitation as a discipline, the mastery of tools and methodologies to evaluate functionally, and the acquisition of knowledge on biomechanics and systems of both analysis and management of information. Therefore, it is necessary a paradigm shift, that begins by promoting training in Rehabilitation from Health Schools, giving space to the learning of biomechanics and methodologies for outcomes measurement to all future physician, regardless of their future specialty, and to other allied health professionals. As for specialists in Physical Medicine and Rehabilitation, it is necessary to standardize the skills and training resources available in relation to biomechanics, functional assessment methodologies, use of new technologies and systems for data analysis and management (31).

To address this, the Panel on Physical Medicine and Rehabilitation of the European Union of Medical Specialists (UEMS) advocates for increasing the subjects related to the specialty in undergraduate training, and proposes the delivery of essential content related to Rehabilitation. As for the competences of the specialist doctor, this association has also developed guidelines that lay the foundations for a harmonized, complete and structured training program, in order to unify criteria and standards in Europe. Among the proposed contents, those related to functional anatomy, biomechanics, and functional assessment within the framework of the International Classification of Functioning (CIF) through the use of clinical methods and instrumental techniques stand out (28). However, not enough importance is yet given to training in new technologies for data management and analysis.

To conclude, and in line with all the above, we consider that **all healthcare professionals (doctors, nurses, etc.)** should have quality didactic resources in at least the following areas of knowledge, always from a practical approach and regarding clinical application:

- Disability and functional assessment within the framework of the ICF. Concepts and methodology.
- Fundamentals of biomechanics: movement and forces. Instrumental techniques for their analysis.
- Basic concepts about biomechanics of gait, balance, spine and most important activities of daily living.
- Main functional assessment tools. Requirements: concepts of validity and feasibility.
- Research methodology. Fundamental concepts on document management, sources of information and statistics in the field of health. Clinical applications of data management systems and artificial intelligence: basic concepts, examples and role in helping diagnosis, decision making or prognosis.

For those professionals working specifically in the rehabilitation field, in addition to the basic knowledge in the areas described, a more in-depth training is proposed, addressing the following aspects:

- Disability and functional assessment according to the ICF. Study of body functions and structures, activity and participation of the person. Concepts and evaluation methodologies.
- Basic physics related to the study of movements, forces, pressures, physiological signals and morphometric parameters. Understanding each parameter.
- Instrumental techniques for its analysis.
   ○ Force and pressure analysis: isokinetic and isometric dynamometers, dynamometric platforms, pressure platforms and blankets, instrumented insoles.
   ○ Movement analysis: electrogoniometers, inertial sensors, 2D/3D and 4D photogrammetry, image recognizing systems based on IA.
   ○ Physiological signals: electromyography, thermography and others.
   ○ Morpho and anthropometric analysis: 3D and 4D scans, systems based on IA.
   ○ New technologies for functional assessment in clinical settings and in the community: wearables and smartphone systems.
- Biomechanics of gait and balance. Normal functioning and main biomechanical alterations in different pathological contexts and considering the activities of daily living. Techniques of analysis and interpretation of results.
- Biomechanics of the spine, upper extremity and lower extremity. Normal functioning and main biomechanical alterations in different pathological contexts and considering the activities of daily living. Techniques of analysis and interpretation of results.
- Concepts on objective functional capacity evaluation. Validity, feasibility, accuracy and responsiveness.
- Concepts of Big Data and Thick Data. Concepts and types of Artificial Intelligence. Application in the clinical field regarding prognosis, prevention, diagnosis and decision-making in Rehabilitation. Ethics and Data Management considerations.
- Specific areas of Rehabilitation and usefulness of biomechanical analysis in the clinical context. Analysis of movements, forces, pressures, physiological signals and/or morfometric parameters, regarding evaluation methodology, interpretation of results and clinical applications in:
   ○ Neurorehabilitation.
   ○ Disorders of the musculoskeletal system.
   ○ Amputees.
   ○ Spinal deformities.
   ○ Others: miscellaneous.

In addition, it would be advisable to establish a network of health centers with infrastructure and resources to accommodate internships in biomechanics and instrumented analysis, and this network should be known and accessible to all future rehabilitation professionals.

## Data Availability

The original contributions presented in the study are included in the article/Supplementary Material, further inquiries can be directed to the corresponding author/s.
